# Exercise, programmed cell death and exhaustion of cardiomyocyte proliferation in aging zebrafish

**DOI:** 10.1242/dmm.049013

**Published:** 2021-07-22

**Authors:** Lindsay B. Murphy, Adrian Santos-Ledo, Tamilvendhan Dhanaseelan, Lorraine Eley, David Burns, Deborah J. Henderson, Bill Chaudhry

**Affiliations:** Biosciences Institute, Faculty of Biomedical Sciences, Newcastle University, International Centre for Life, Central Parkway, Newcastle upon Tyne NE1 3BZ, UK

**Keywords:** Cardiomyocyte turnover, Fibrosis, Aging, Zebrafish heart, Exercise

## Abstract

Exercise may ameliorate the eventual heart failure inherent in human aging. In this study, we use zebrafish to understand how aging and exercise affect cardiomyocyte turnover and myocardial remodelling. We show that cardiomyocyte proliferation remains constant throughout life but that onset of fibrosis is associated with a late increase in apoptosis. These findings correlate with decreases in voluntary swimming activity, critical swimming speed (Ucrit), and increases in biomarkers of cardiac insufficiency. The ability to respond to severe physiological stress is also impaired with age. Although young adult fish respond with robust cardiomyocyte proliferation in response to enforced swimming, this is dramatically impaired in older fish and served by a smaller proliferation-competent cardiomyocyte population. Finally, we show that these aging responses can be improved through increased activity throughout adulthood. However, despite improvement in Ucrit and the proliferative response to stress, the size of the proliferating cardiomyocyte population remained unchanged. The zebrafish heart models human aging and reveals the important trade-off between preserving cardiovascular fitness through exercise at the expense of accelerated fibrotic change.

## Abstract (precis)

**Summary:** A zebrafish model for studying how aging and exercise affect cardiomyocyte turnover in the human heart reveals a decrease in the proliferative capacity of the heart, which is not prevented by exercise.

## INTRODUCTION

Cardiomyocytes are subjected to continuous mechanical and biochemical stresses which contribute to the physiological aging process (Sheydina et al., 2011; Shao et al., 2020). This decline in basal and peak cardiac performance during normal human chronological age is linked with cardiomyocyte loss, deposition of extracellular matrix and adverse myocardial remodelling. It is now recognised that these are secondary to accumulating failures of a wide range of cellular processes, including mitochondrial dysfunction and management of oxidative stress (Chiao and Rabinovitch, 2015; Obas and Vasan, 2018; Shao et al., 2020). These decreases in cardiac performance are initially only recognised if they limit voluntary activities. However, progression of the aging cardiomyopathy eventually leads to diastolic dysfunction, the clinical manifestations of heart failure and increasing risk of arrythmia (Lakatta, 2002). Several lifestyle strategies have been proposed to alleviate this decline and promote healthy longevity, including calorie restriction and exercise (Berthelot et al., 2019). Regular aerobic exercise is considered to improve cardiac outcomes in this group (Banks et al., 2016), but an age-related decline in the ability to perform exercise is also well recognised (Gava and Ravara, 2019). The relative contributions of physiological, behavioural or other factors in this declining activity is unknown, and it is unclear whether reduced cardiovascular function is a consequence of reduced activity or vice versa (Baker and Tang, 2010). Importantly, the heart also needs to be able to acutely increase pumping activity in response to a wide range of situations, including injury and infections. Previous studies have shown that throughout life the myocardium exhibits low rates of proliferation, which are concentrated in the mononuclear diploid cardiomyocyte component (Becker and Hesse, 2020). In contrast to other organs, classical stem cell populations do not reside within the heart. Instead, proliferation occurs from pre-existing cardiomyocytes (Bo et al., 2021). However, it is not known whether cardiomyocyte proliferation is responsive to physiological stress or whether this alters with age.

A wide range of laboratory aging models, ranging from *Drosophila* (Blice-Baum et al., 2019) to mice (Cohen, 2018), have been used to understand fundamental conserved principles of aging. However, lifespan and the aging phenotype are not universally correlated across different animal species. The Hamiltonian pattern of aging (Cohen, 2018) and the inverted U-curve of physical performance (Berthelot et al., 2019) both describe an accelerating rate of decline beginning after sexual maturity. This is characteristic of human aging and an aging pattern also found in mammals, avians and other animals, including bony fish (Cohen, 2018). Hence, zebrafish have been suggested as a model for aging studies (Gerhard and Cheng, 2002; Kishi, 2014; Herrera and Jagadeeswaran, 2004). Laboratory zebrafish generally have a life span of ∼36 months (Herrera and Jagadeeswaran, 2004; Gerhard et al., 2002), although this may vary slightly with different strains or sources (Gerhard et al., 2002). It has been suggested that zebrafish exhibit indeterminate growth (Gerhard et al., 2002; Singleman and Holtzman, 2012) but paradoxically also display aging-related degenerative changes, including scoliosis (Gerhard et al., 2002). However, other studies have questioned this indeterminate growth pattern (Sun et al., 2015; Biga and Goetz, 2006). Furthermore, it is not known whether the zebrafish heart has an indeterminate growth pattern, although it is recognised that the myocardial response to physiological stimulus is predominantly through hyperplasia (Sarantis et al., 2019) rather than hypertrophy (Hendricks et al., 2009; Zhou et al., 2020). Taken together, zebrafish would appear to be a tractable model for studying the proliferative response of cardiomyocytes during normal aging and in response to physiological stimuli, such as exercise.

In this study, we set out to establish the natural history of adult zebrafish heart growth from sexual maturity to old age by evaluating cardiomyocyte proliferation, apoptosis and myocardial remodelling, and compare this with their cardiovascular fitness determined through analysis of spontaneous and maximal swimming activity in young and middle-aged fish. We then evaluated the effect of short-term intense exercise on cardiomyocyte turnover in these age groups and finally sought to understand the extent to which cardiomyocyte turnover, myocardial architecture and the hyperplasia response to stress are affected by long-term sustained lower intensity exercise. Using dual-pulse labelling with thymidine analogues, we were also able to identify altered patterns of cardiomyocyte proliferation following physiological stress in older fish.

## RESULTS

### Somatic and ventricular growth diminishes with age in zebrafish

There are conflicting views regarding indeterminate somatic growth in zebrafish (Gerhard et al., 2002; Sun et al., 2015; Biga and Goetz, 2006), and it is not known whether indeterminant growth extends to the heart. To address these questions, we examined the length of the body and the cardiac ventricle in groups of wild-type laboratory zebrafish, culled at intervals from 6 months of age, when fully sexually mature, until 36 months of age, which represents old age for inbred laboratory zebrafish strains (Herrera and Jagadeeswaran, 2004; Gerhard et al., 2002).

**Fig. 1.**
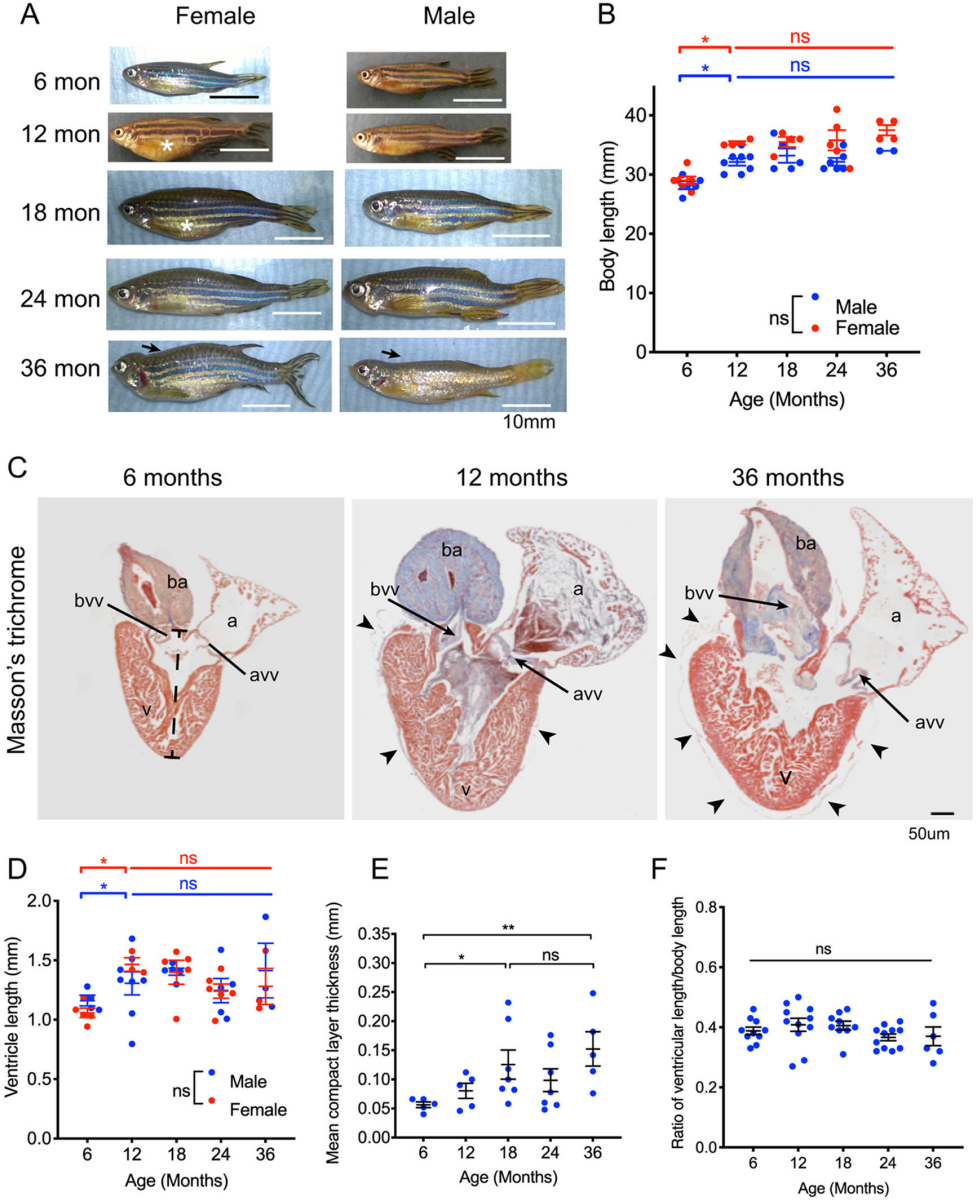
**Zebrafish do not exhibit indeterminate somatic or ventricular growth.** (A) External appearances of male and female zebrafish between 6 months and 36 months of age. Female fish have larger abdomen than male fish (asterisk). Spinal deformities are more common and increasingly severe after 24 months (arrows). (B) Lengths of male (blue points) and female (red points) zebrafish with increasing age. Sample size (*n*) is 11, 11, 10, 11, 6 for age groups 6, 12, 18, 24 and 36 months, respectively. There were no differences between the lengths of male and female fish at any stage. After a rapid increase in length in the first 6 months, there was a smaller increase in length in the second 6 months of life, but no change thereafter. (C) Midline histological sections through zebrafish hearts stained with Masson's trichrome. Myocardium is indicated in brown and collagen in blue. The 6-month-old heart had minimal collagen staining in the bulbus arteriosus (ba). Epicardial adipose tissue is not seen. At 12 months, the heart was larger and the bulbus stained strongly for collagen fibres, and there is some epicardial adipose tissue (arrowheads). In the 36-month-old heart, there were extensive epicardial adipose deposits seen on the surface of the heart (arrowheads). There were also extensive collagen deposits in the bulbus arteriosus (ba), bulbo-ventricular valve (bvv) and atrioventricular valve (avv). (D) Ventricular length measured from the histological sections in C, from apex to base of chamber, indicated by the dashed bar in C (6 months). All individual data points are shown (male, blue points; female, red points). Sample size (*n*) was 11, 11, 10, 11 and 6 for age groups 6, 12, 18, 24 and 36 months, respectively. Ventricular length increased between 6 and 12 months but not thereafter, and there was no difference between hearts from male and female fish. (E) Mean thickness of the ventricular myocardium compact layer increased until 18 months of age and did not change thereafter. Sample size (*n*) was 5, 5, 7, 7 and 5 for age groups 6, 12, 18, 24 and 36 months, respectively. (F) Ratio of ventricular length (D) to body length (B). Sample size (*n*) is 10, 11, 9, 11 and 6 for age groups 6, 12, 18, 24 and 36 months, respectively. There was no difference in the ratio throughout adult life. All individual data points are shown. Data are mean±s.e.m. *P<0.05; **P< 0.02; ns, not significant (two-way ANOVA was used in B,D; one-way ANOVA in E,F). a, atrium; avv, atrioventricular valve; ba, bulbus arteriosus; bvv, bulbo-ventricular valve; v, ventricle.

During the first 12 months of life, both male and female zebrafish grew rapidly with increasing body length (Fig. 1A,B). Although female fish appeared to have larger abdomen than male fish, there was no difference in length (Fig. 1A,B). From 12 months of age, both male and female fish stopped increasing in length, and by 24 months, fish were beginning to show degenerative changes, such as scoliosis (Gerhard et al., 2002; Fig. 1A). To understand how overall cardiac growth changes with age, we measured the size of the ventricle in these different-aged fish (Fig. 1C-F). As with somatic length, the ventricular length increased from 6-12 months but remained unchanged after this (Fig. 1C,D), and there was no difference between male or female fish (Fig. 1D). As well as the overall length of the ventricle, we measured the thickness of the outer compact layer of the ventricular myocardium in midline histological sections. There was a gradual thickening with age but after 18 months of age no increase in thickness could be detected (Fig. 1E). Finally, we evaluated the relative size of the heart to body by calculating the ratio of ventricular length to body length (Fig. 1F). There was no difference in this ratio across the adult ages. Thus, although zebrafish exhibit early rapid somatic growth, it is not indeterminant. Similarly, although the heart initially increases in overall size and there is myocardial thickening, this also ceases in late adulthood, and therefore, the zebrafish heart does not exhibit indeterminant growth.

### Cardiac fibrosis increases with age

**Fig. 2.**
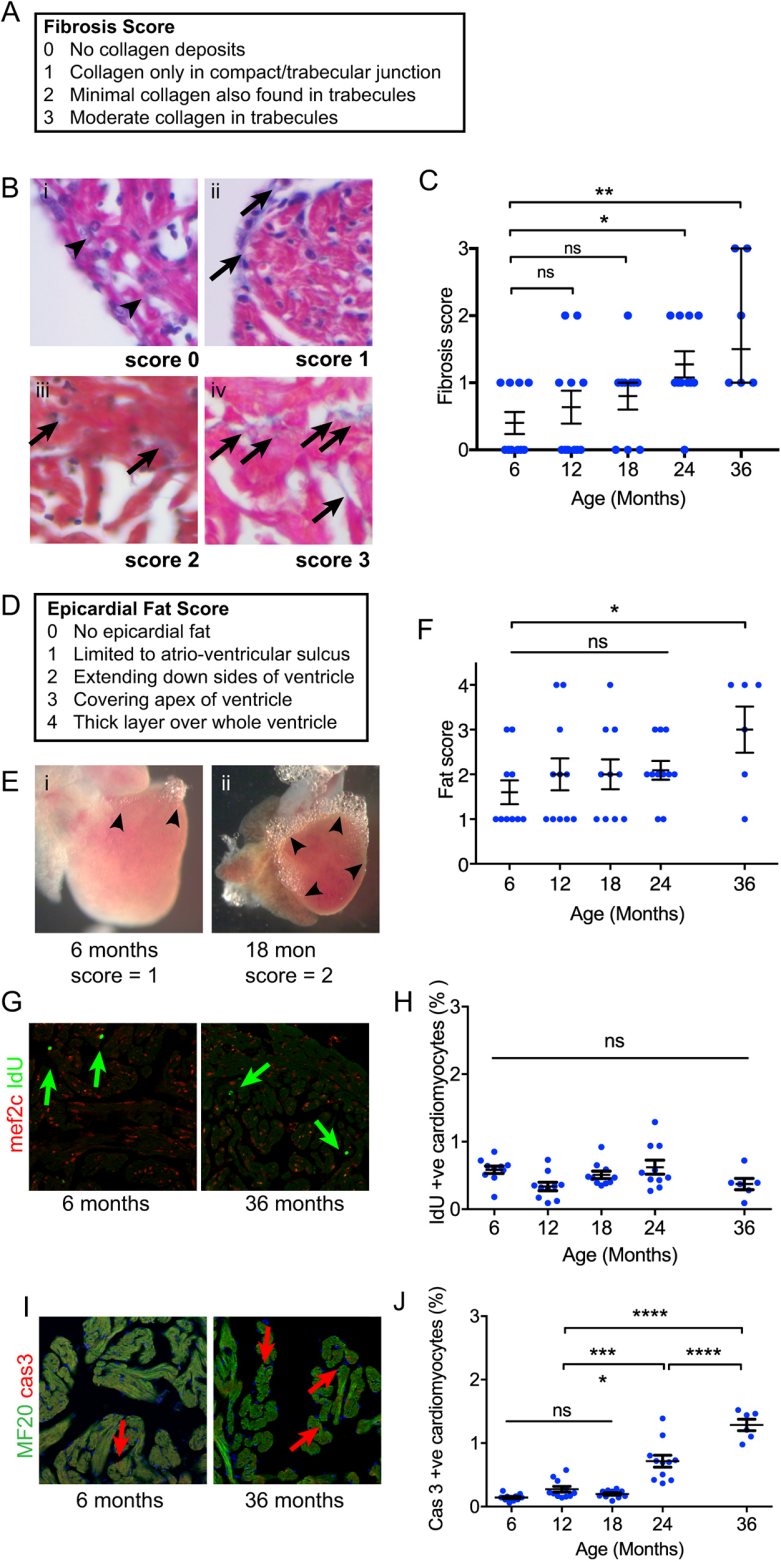
**Myocardial histology and cardiomyocyte turnover with aging.** (A) Description of semiquantitative fibrosis score. (B) Appearances of each fibrosis score category. Arrowheads (i) indicate the absence of collagen at the compact-trabecular junction. Arrows indicate deposits of fibrosis at the compact-trabecular junction (ii) and in the trabecular myocardium (iii and iv). (C) Fibrosis score with age. Sample size (*n*) was 10, 11, 10, 11 and 6 for age groups 6, 12, 18, 24 and 36 months, respectively. All individual data points are shown with median and quartiles. The median fibrosis score was elevated at 24 and 36 months (Kruskal–Wallis test, non-parametric ANOVA). (D) Description of semiquantitative epicardial fat score. (E) Epicardial fat limited to the atrioventricular sulcus (i) and extending over sides (ii). (F) Increased epicardial fat score at 36 months (one-way ANOVA). Sample size (*n*) was 10, 11, 10, 11 and 6 for age groups 6, 12, 18, 24 and 36 months, respectively. (G) Examples of immunofluorescence labelling of IdU incorporation (arrows) at 6 and 36 months. (H) Percentage of cardiomyocytes incorporating IdU remains constant with age. Sample size (*n*) was 10, 10, 10, 10 and 6 for age groups 6, 12, 18, 24 and 36 months, respectively. (I) Nuclei labelling with activated caspase 3 antibodies (arrows). (J) Increased frequency of caspase^+^ cardiomyocyte nuclei from 24 months of age (one-way ANOVA). Sample size (*n*) was 10, 11, 10, 11 and 6 for age groups 6, 12, 18, 24 and 36 months, respectively. All individual data points are shown. Data are mean±s.e.m. *P<0.05; **P<0.02; ***P<0.001; ****P<0.0001; ns, not significant.

In human hearts, diffuse fibrosis appears with aging. We therefore examined midline sections of zebrafish hearts for evidence of collagen deposition using Masson's trichrome and a semiquantitative fibrosis scoring system (Fig. 2A). In contrast to the heart of mammals and birds in which fibroblasts are distributed throughout the myocardium, the zebrafish heart exhibits a single layer of fibroblasts lying between cortical and trabecular myocardial layers (Lafontant et al., 2013). At 6 months of age, a thin layer of collagen fibres between the compact and trabecular layers was noted (Fig. 2A,B). Although this did not become more prominent with age (Fig. 2C), diffuse fibrosis associated with the trabeculae of the ventricles was also found at 24 months, and this was more evident by 36 months (Fig. 2B,C).

### Epicardial adipose tissue

At all ages, there were varying amounts of adipose tissue deposited on the surface of the heart (Fig. 2D-F). In some fish, fat was found to be confined to the atrioventricular sulcus and the bulbar ventricular junction but was scarce across the bulk of the ventricle, and in others, this extended to completely cover the ventricular apex (Fig. 2E). In the oldest fish, there was a thick layer of fat (Fig. 2E,F).

### Cardiomyocyte cycling remains constant but programmed cell death increases in the aging zebrafish heart

The cessation of linear somatic and cardiac growth associated with degenerative changes in the myocardial architecture prompted us to investigate whether cardiomyocyte turnover was also altered with aging. Cardiomyocytes in the adult zebrafish are almost all mononuclear and diploid (Patterson et al., 2017), and so the incorporation of thymidine analogues during DNA synthesis can be equated with cell proliferation. We exposed groups of zebrafish at different ages to iododeoxyuridine (IdU)-containing water for 24 h and identified incorporation into nuclear DNA. Cardiomyocyte nuclei were identified using an antibody against Mef2c (Fig. 2G). In all age groups, from 6 to 36 months, the frequency of IdU-labelled cardiomyocytes remained unchanged at ∼0.5%, indicating constant low level cardiomyocyte proliferation throughout adult life (Fig. 2H). Programmed cell death was evaluated using antibodies to detect activated-caspase 3 on adjacent tissue sections (Fig. 2I,J). For this experiment, cardiomyocytes were identified by immunolabelling with MF20 antibody to avoid cross reactivity with activated-caspase 3 antibodies (Fig. 2I). Although only 0.1% of cardiomyocytes were undergoing apoptosis between 6 and 18 months of age, at 24 months the rate of programmed cell death increased to 0.8% and to 1.4% by 36 months (Fig. 2J). These proliferation and programmed cell death indices, in conjunction with the histological studies, do not support indeterminant growth but instead a pattern of initial growth with later increased cell death and fibrotic remodelling.

### Physiological consequences coincident with cardiac aging

**Fig. 3.**
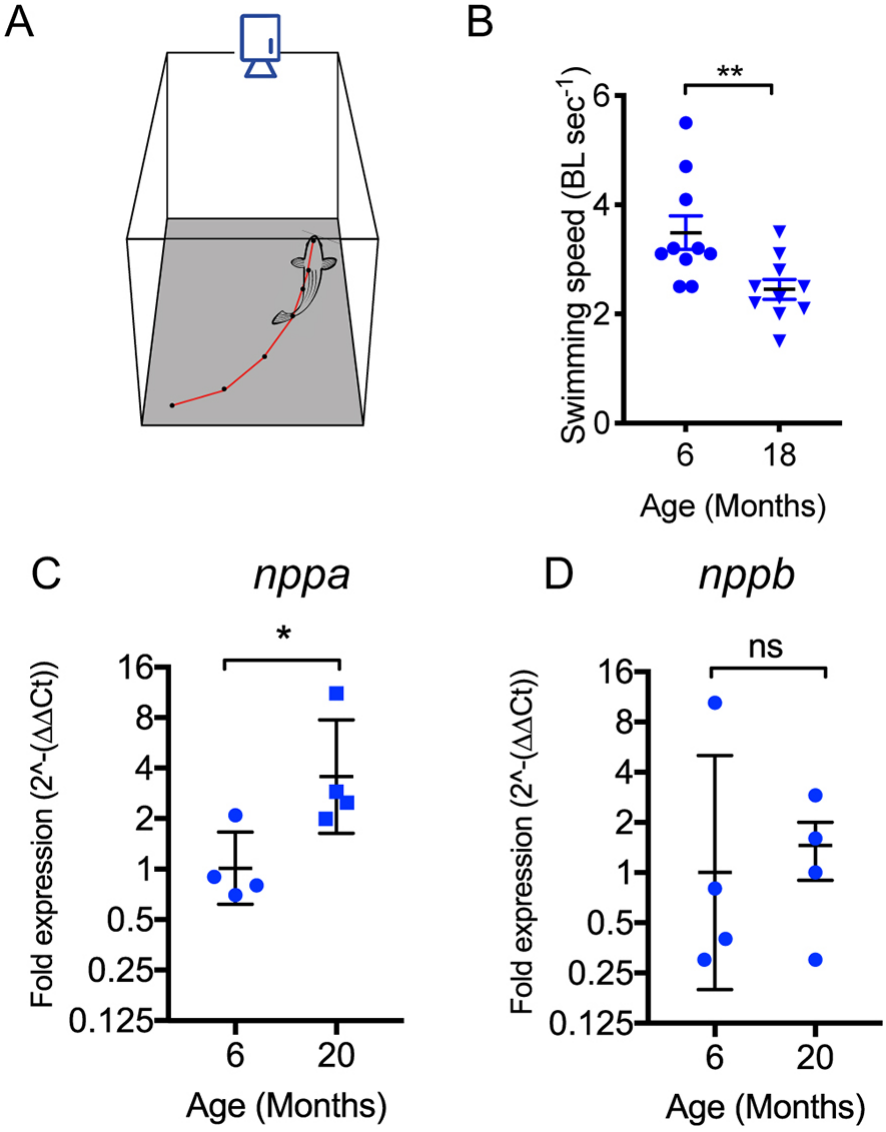
**Physiological effects of aging in zebrafish.** (A) Experimental setup to assess voluntary swimming. Individual fish were placed in a 3-litre tank, with white paper below and digital video captured from above. The position of the head was tracked manually using Fiji/ImageJ. (B) Voluntary swimming speeds in body lengths per second for 6-month and 18-month-old zebrafish. Sample size (*n*) was ten for both 6- and 18-month age groups. (C,D) Quantitative RT-PCR analysis of *nppa* and *nppb* gene expression in whole hearts. Sample size (*n*) was ten for both 6- and 18-month age groups. All individual data points are shown, mean±s.e.m. Normal distribution of data (D'Agostino–Pearson), no difference in variance (*F*-test). *P<0.05; **P<0.02; ns, not significant (unpaired two-tailed Student's *t*-test).

We next investigated whether this period of adverse remodelling from 24 months, due to elevated levels of programmed cell death, was related to a previous change in physical activity. To evaluate this, the spontaneous swimming behaviour of mature zebrafish at 18-months of age was compared to that of a group aged 6 months, using digital video recording and path tracking (Fig. 3A). In the 6-month-old fish, the mean spontaneous swimming speed was 3.25 BL sec^−1^ (body lengths per second), but only 2.5 BL sec^−1^ in the 18-month-old zebrafish (Fig. 3B). To help identify whether this difference in spontaneous swimming activity between the 6- and 18-month-old zebrafish was related to changes in cardiovascular function rather than altered behaviour, we measured *nppa* and *nppb* transcripts in whole hearts by qRT-PCR. These atrial (ANP) and brain (BNP) natriuretic factors are evolutionarily conserved biomarkers of cardiac stress produced by the natriuretic peptide precursor A (*nppa*) and B (*nppb*) genes, and elevations in the activation of these genes is suggestive of physiological cardiac decompensation (Man et al., 2018). The levels of *nppa* transcripts were 4-fold higher in 20-month-old fish compared to 6-month-old fish (Fig. 3C). However, there was no change in the levels of *nppb* transcripts (Fig. 3D). This was not surprising as the *nppb* gene responds less strongly to stress than does *nppa* (Man et al., 2018), suggesting that at 18- to 20-months of age the zebrafish cardiovascular system is stressed and at a stage of impending decompensation.

### Critical swimming speed diminishes with age

**Fig. 4.**
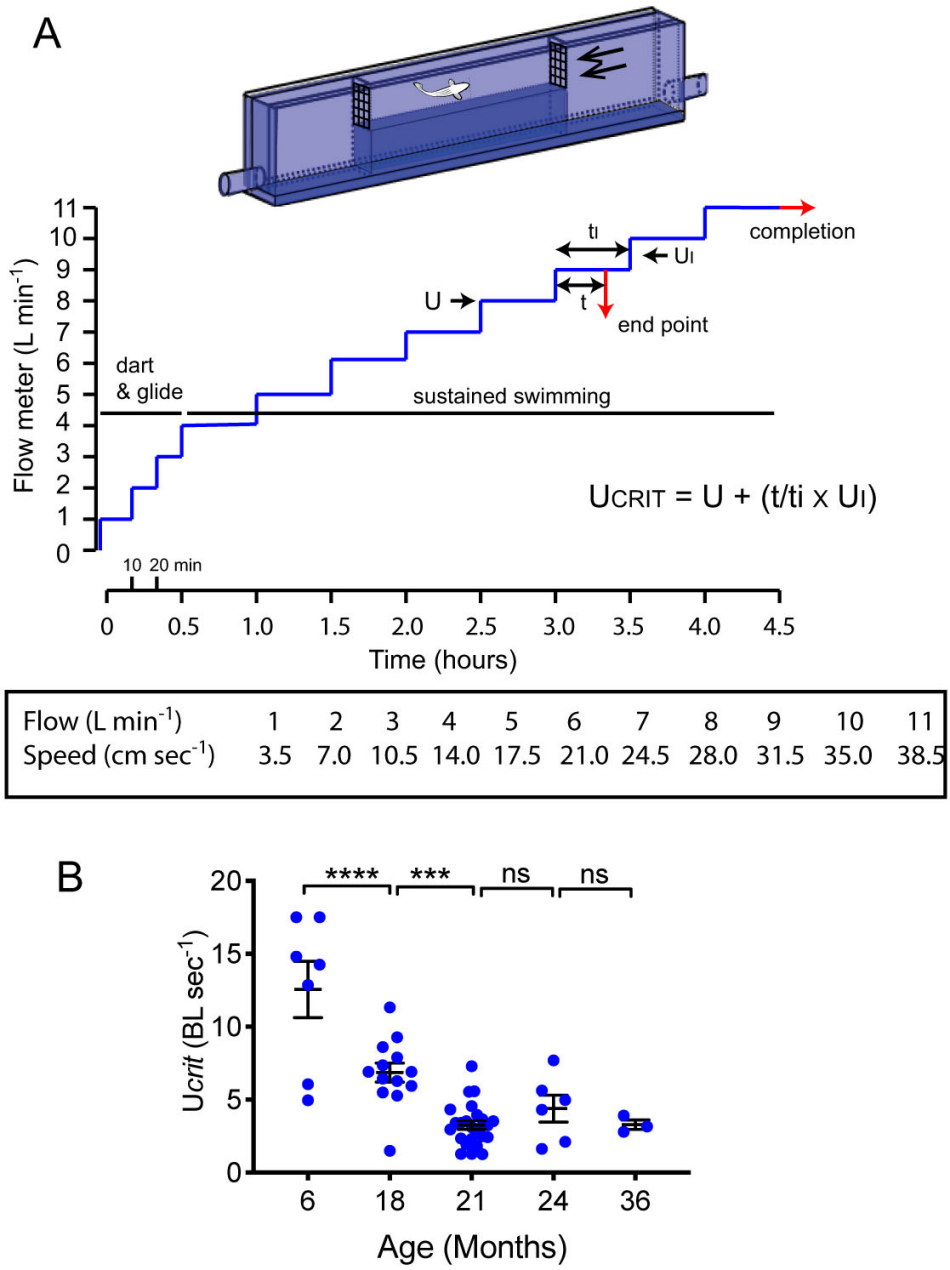
**Critical swimming speed diminishes with aging.** (A) Critical swimming speed protocol. Individual fish were placed in a modified flume containing a narrow cross-section to increase water speed. During the first 30 min, the water speed is increased to 14 cm sec^−1^ to allow acclimatisation and allow transition from dart and glide to sustained swimming patterns. When the fish were unable to maintain swimming speed, the water speed of the last completed stage, with allowance for proportion of current stage, was used to calculate Ucrit. A flow meter (L min^−1^) was used to follow increases in water speed (cm sec^−1^). (B) Progressive fall in Ucrit from 6 to 21 months (one-way ANOVA). All individual data points are shown, Data are mean±s.e.m. Sample size (*n*) was 7, 13, 26, 6 and 3 for age groups 6, 18, 21, 24 and 36 months, respectively. ***P<0.001; ****P<0.0001; ns, not significant.

To objectively evaluate whether this reduced spontaneous activity was associated with an age-related reduction in overall exercise capability, rather than behaviour, we measured the critical swimming speed of individual fish at stages between 6 and 36 months of age in a purpose-built flume (Fig. 4). The protocol began with a period of acclimatisation and increase in flow to allow the transition from dart and glide to constant swimming activity. Zebrafish were then subjected to an increasing water speed in 30-min incremental steps until they were no longer capable of maintaining their position in the water flow. The range of water flows were chosen to ensure that swimming activity represented aerobic rather than anaerobic exercise capacity, and that there would be sufficient opportunity to discriminate between different exercise capabilities (Fig. 4A). These experiments showed an age-related fall in critical swimming speed. At 6 months of age, the mean Ucrit was 12.6 BL sec^−1^, but this fell to 6.9 BL sec^−1^ at 18 months and 3.3 BL sec^−1^ at 21 months. At 24 and 36 months of age, the Ucrit remained similar to 21 months of age. Thus, 6-month-old zebrafish were capable of mounting a 700% increase in swimming speed, as measured through Ucrit (Fig. 4B), compared to that observed during spontaneous swimming (Fig. 3B). In contrast, from 21 months of age, the fish could only maximally increase their swimming speed by 60% (Fig. 3B, Fig. 4B).

Taken together, these studies indicate an age-related reduction in spontaneous and maximal swimming activity, with elevated biomarkers indicating early cardiac dysfunction. These changes appear before a marked increase in the level of cardiomyocyte apoptosis, suggesting that apoptosis and subsequent fibrosis may be responses to initial myocardial dysfunction.

### Short-term high-intensity swimming in 21-month-old zebrafish reveals cardiomyocyte proliferative exhaustion

**Fig. 5.**
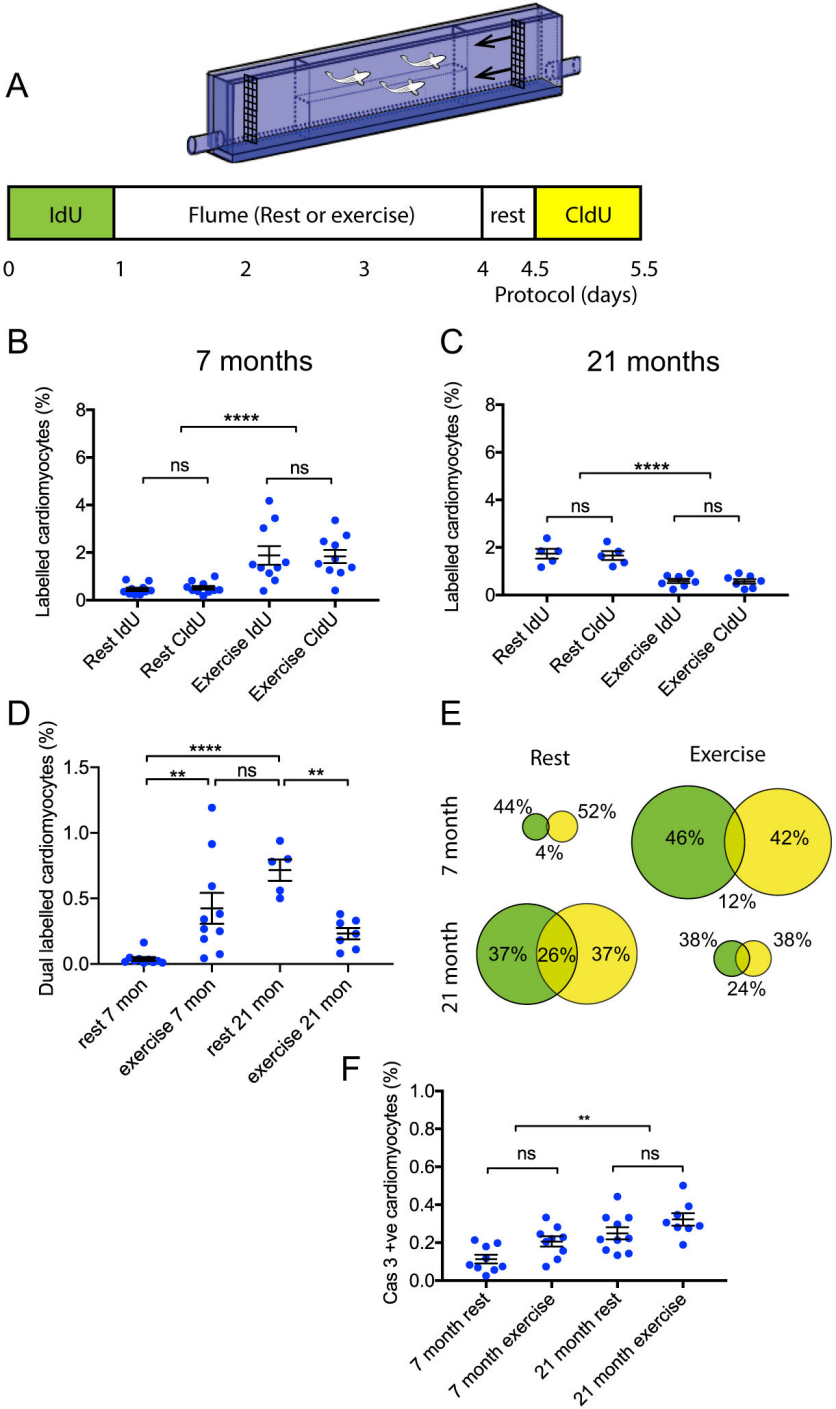
**Cardiomyocyte turnover in response to 3-day high-intensity swimming stress test.** (A) Experimental protocol. Zebrafish were first exposed to water containing IdU for 24 h. They were then placed in the Ucrit flume, modified to house groups of fish at moderate water flow by removing the narrow channel insert. The exercise group were placed in running water at 3 BL sec^−1^ for 72 h. The control groups were placed in an identical flume with no water flow (rest conditions). After 72 h in the flumes, all fish received 12 h of rest in holding tanks and were exposed to CldU for 24 h, and then culled. (B) Percentage of cardiomyocytes labelling with IdU or CldU in 7-month-old fish under high-intensity swimming (*n*=10) or rest conditions (*n*=10). (C) Percentage of cardiomyocytes labelled with IdU or CldU in 21-month-old fish under high-intensity swimming (*n*=7) or rest conditions (*n*=5). (D) Percentage of cardiomyocytes dual labelled with IdU and CldU in 7-month-old and 21-month-old zebrafish under rest and high-intensity swimming conditions. Sample numbers as in B and C. (E) Venn diagrams summarising CldU and IdU results. Diameter of circles indicates relative magnitude of thymidine analogue incorporation. Percentages indicate proportions of cycling cardiomyocytes within each group. (F) Percentage of cardiomyocytes immunolabelling with activated caspase 3 antibodies in 7-month-old zebrafish under rest (*n*=9) or exercise (*n*=8), and 21-month-old zebrafish under rest (*n*=10) or exercise (*n*=8) conditions. Data are mean±s.e.m. **P<0.02; ****P<0.0001; ns, not significant (one-way ANOVA).

Exercise is considered to be beneficial to the aging heart (Banks et al., 2016; Gava and Ravara, 2019), and we wondered whether this benefit might occur through induction of cardiomyocyte replication or suppression of apoptosis. To examine this, we modified the purpose-built flume to house groups of 7-month-old and 21-month-old zebrafish for 72 h in water running at a speed of 3 BL sec^−1^ (Fig. 5). This swimming speed was chosen because it was close to the mean Ucrit value for 21-month-old fish (3.3 BL sec^−1^), suggesting that the continuous water flow should optimally challenge this group. The zebrafish were first pulsed with IdU for 24 h, allowed to swim in the flume for 72 h, and then pulsed with chlorodeoxyuridine (CldU) for 24 h. An identical protocol was provided for groups of control fish in a second identical flume with static water conditions. Hearts were sectioned and labelled with antibodies to separately detect IdU and CldU, or alternatively, activated-caspase 3 expression. In both cases, cardiomyocytes were identified by labelling with an antibody raised against either Mef2c or MF20 as described previously. Notably, there was no wash out period between exposure to IdU and commencement of the swimming challenge (Fig. 5A). Hence, IdU continued to be incorporated into cardiomyocytes during the early phase of the challenge and reflected the immediate response of the cardiomyocytes to high-intensity exercise. In contrast, the CldU exposure would indicate cell replication occurring 3 days later at the end of the exercise period.

The 7-month-old zebrafish placed within the flume under resting conditions exhibited a low level of cardiomyocyte proliferation (∼0.5%), which was in keeping with our previous data (Fig. 2H), and those 7-month-old fish that undertook the high-intensity swimming demonstrated a 3.8-fold increase in the incorporation of both IdU and CldU (Fig. 5B). Unexpectedly, when the 21-month-old fish were placed within the flume under static water conditions, there were elevated levels of IdU and CldU incorporation affecting ∼2% of cardiomyocytes (Fig. 5C). This was much higher than that seen at 18 (0.5%) and 24 months of age (0.6%), and is likely to reflect a response to the stress of being placed within the flume. When 21-month-old fish were exposed to the high-intensity swimming regimen, they demonstrated very low levels of both IdU and CldU incorporation at 0.2% (Fig. 5C). This was lower than their static flume counterparts, but also lower than other age-matched controls (Fig. 2H), potentially indicating not only an inability to mount a replicative response, but also an exhaustion of homeostatic capability.

The delivery of a CldU pulse 3 days after IdU also allowed us to identify cardiomyocytes that had continued to replicate throughout this period, as opposed to cells that completed DNA synthesis either during initial exposure to IdU or alternatively after later exposure to CldU (Fig. 5D,E). Most of the cardiomyocyte replication in spontaneously swimming 7-month-old fish, occurred in different cardiomyocytes, and only 4% of the replicating cardiomyocyte population incorporated both thymidine analogues. Although the absolute percentage of cardiomyocytes replicating increased upon exercise in the 7-month-old fish (Fig. 5B), there was also more than a 12% increase in the proportion of dual-labelled cardiomyocytes (Fig. 5D,E). In contrast, although the 21-month-old fish exhibited elevated basal levels of proliferation when placed in the static flume, and a much-reduced level of proliferation upon exercise, the proportion of dual-labelled cardiomyocytes remained at ∼25% in both situations. Despite these dynamic proliferative responses, there were no changes in the levels of programmed cell death in either exercising or static water groups at either age (Fig. 5F).

Taken together, these proliferative responses suggest that basal cardiomyocyte proliferation in young fish generally involves different cardiomyocytes entering and completing cell cycle, with relatively few cells being stimulated to continue cycling when stressed. However, in older fish it appears that the pool of cardiomyocytes able to enter cell cycle is reduced and consequently a larger proportion of continually cycling cells are evident. Under severe stress conditions, the total cycling population is exhausted.

### Prolonged exercise increases fitness but also exacerbates cardiac fibrosis

**Fig. 6.**
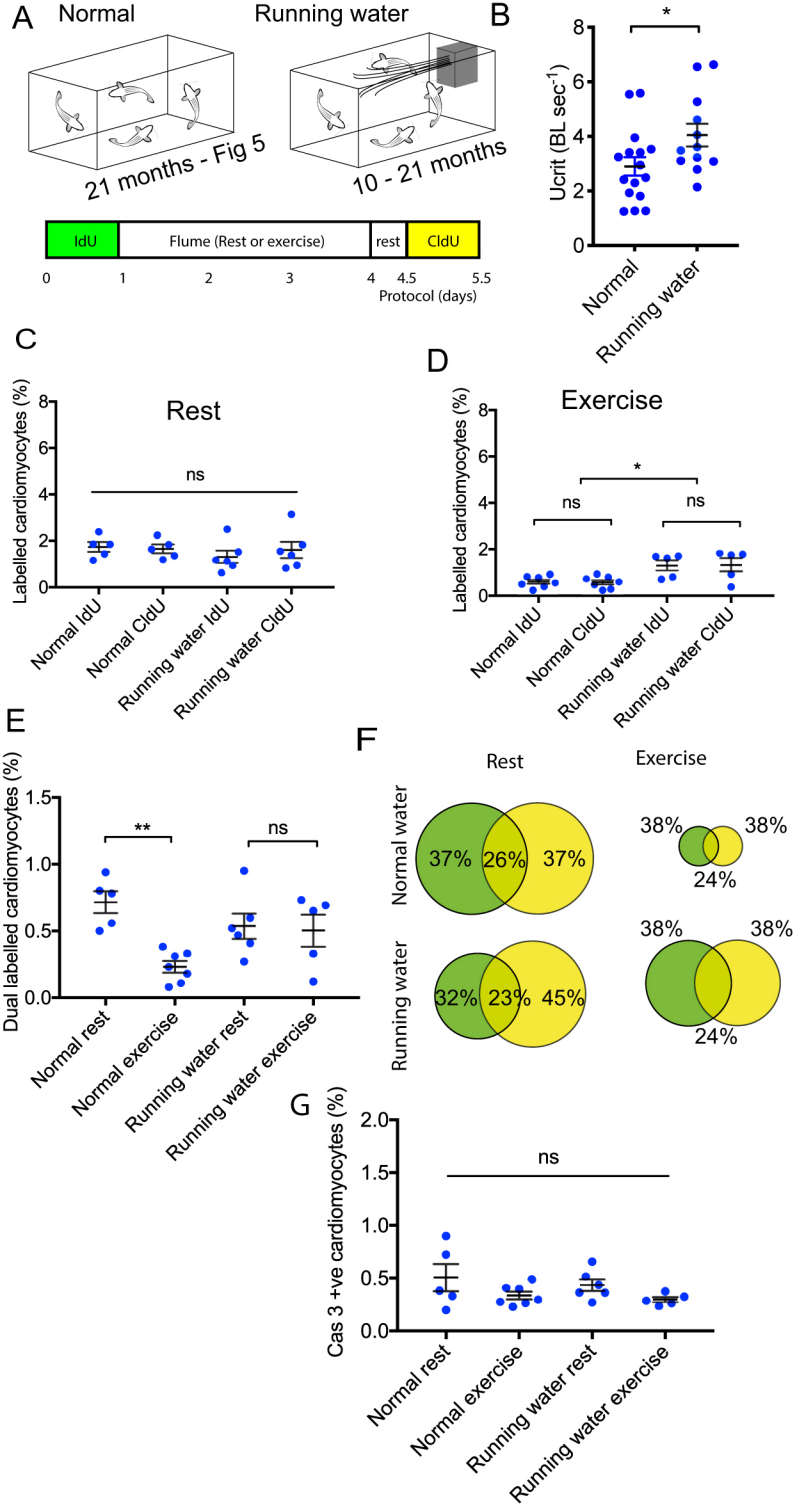
**Cardiomyocyte turnover in response to 3-day high-intensity swimming stress test after being raised in running water.** (A) Experimental protocol. Data from fish raised under normal water conditions (normal) are reproduced from Fig. 5 for ease of comparison. Long-term running water conditions were produced by placing an 8-litre min^−1^ impeller pump/filter unit inside a holding tank containing siblings of fish described in Fig. 5 when 10 months of age. Both normal and running water conditions zebrafish were given the 3-day high-intensity swimming stress test (see Fig. 5), and critical swimming speed was then determined (see Fig. 4). (B) Critical swimming speed of zebrafish raised in normal (*n*=15) and in running water conditions (*n*=12) at 21 months. No difference in variance. (C) IdU and CldU incorporation into cardiomyocytes for zebrafish raised in normal conditions (*n*=5) and in running water (*n*=6) with the 3-day high-intensity swimming protocol under rest conditions. (D) IdU and CldU incorporation into cardiomyocytes for zebrafish raised in normal (*n*=7) and running water (*n*=5) with the 3-day high-intensity swimming protocol under exercise conditions. (E) Percentage of cardiomyocytes dual labelled with both IdU and CldU under rest or exercise conditions for zebrafish raised in normal or running water conditions. Subject numbers as in C and D. (F) Venn diagrams summarising CldU and IdU results. Diameter of circles indicates relative magnitude of thymidine analogue incorporation. Percentages indicate proportions of cycling cardiomyocytes within each group. (G) Percentage of cardiomyocyte immunolabelling with activated caspase 3 antibodies in 21-month-old zebrafish raised in normal conditions under rest (*n*=5) or exercise (*n*=7), and for zebrafish raised in running water conditions under rest (*n*=6) or exercise conditions (*n*=5). Data are mean±s.e.m. Statistical significance was determined using an unpaired two-tailed Student's *t*-test (B) and one-way ANOVA (C-E,G). *P<0.05; **P<0.02; ns, not significant.

**Fig. 7.**
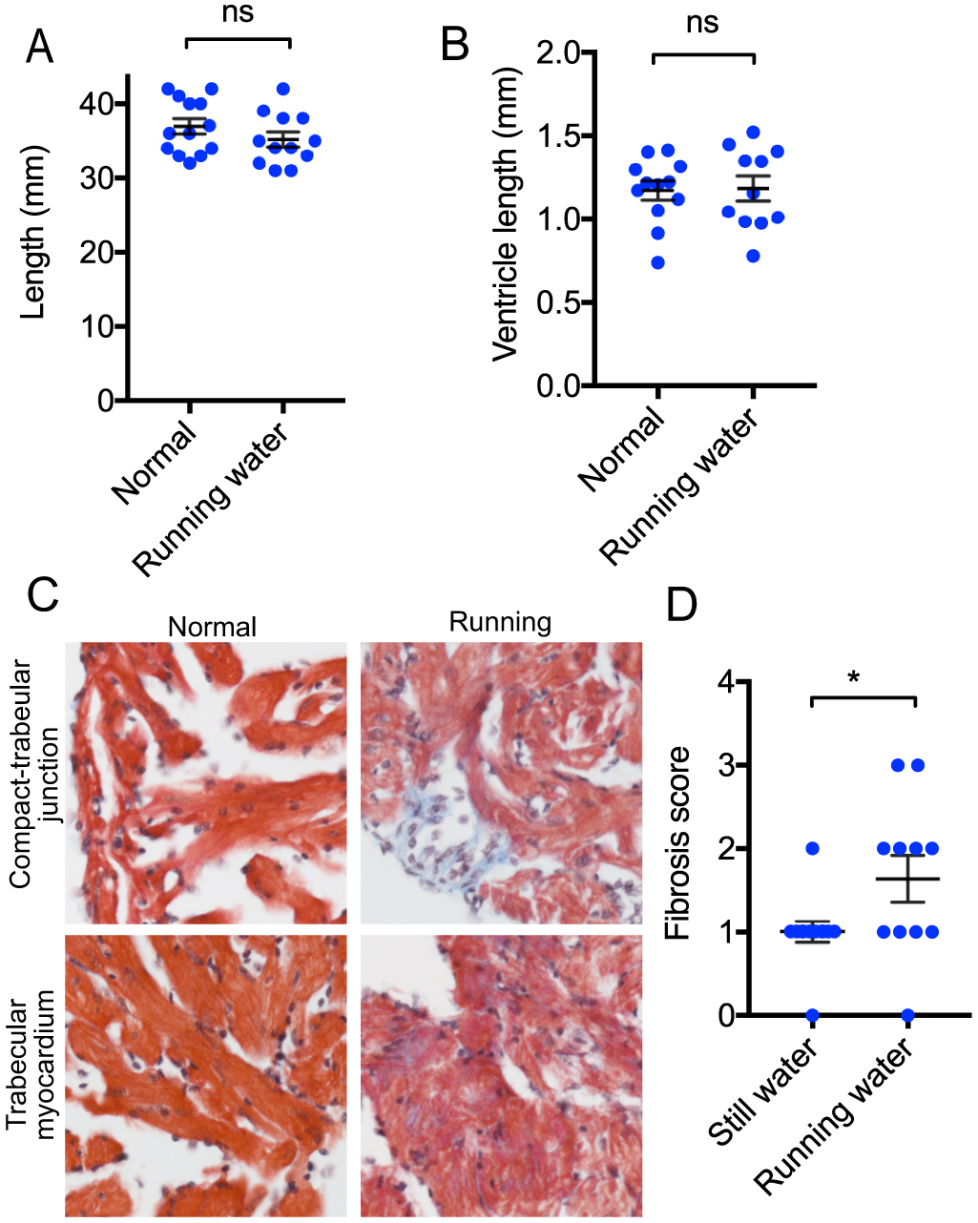
**Increased cardiac fibrosis after prolonged exercise.** (A) Body length in 21-month-old fish raised in either normal (*n*=13) or running water (*n*=12). (B) Ventricle length in 21-month-old fish raised in either normal (*n*=12) or running water (*n*=11). (C) Increased myocardial fibrosis in zebrafish maintained in running water conditions. (D) Myocardial fibrosis scores in zebrafish maintained in running water conditions (*n*=12) or running water (*n*=11). Data are mean±s.e.m. *P<0.05; ns, not significant (unpaired two-tailed Student's *t*-test).

The exhaustion of cardiomyocyte proliferation in the 21-month-old fish undertaking high-intensity swimming was an unexpected finding. As young zebrafish demonstrate greater levels of spontaneous activity than older fish (Fig. 3), we investigated whether improving the cardiovascular fitness of zebrafish, through the high-intensity exercise challenge of a long-term increase in their swimming activity, could change their cardiomyocyte response. To increase their basal activity, we installed a small aquarium impeller pump within a 12-litre holding tank to create a stream of 2 BL sec^−1^ through the centre of the tank. This provided a strong flow that the fish would need to navigate during normal feeding and behaviour, but also permitted low flow areas to exist to allow fish to rest. Zebrafish were introduced to the tank at 10 months of age and were examined at the end of the 11-month period in running water (Fig. 6A). Because of the length of these experiments and to reduce the numbers of animals used, their 21-month-old siblings raised in normal still water conditions, and initially presented in Fig. 5, acted as controls, and their data are reproduced in Fig. 6 for convenience. Twenty-one-month-old zebrafish, raised in running water conditions for 11 months, were then introduced to the 72-h high-intensity swimming experiment, with half the group maintained in static flume conditions and half being placed in water flowing at 3 BL sec^−1^ for 72 h (Fig. 6A). At the end of the experiment, the critical swimming speeds of both groups were assessed. Zebrafish raised in running water had a significantly greater exercise capacity and achieved 38% greater Ucrit values than their siblings raised in still water (Fig. 6B). Cardiomyocyte proliferation through IdU and CldU incorporation was assessed as before. The 21-month-old fish raised in running water and their siblings raised in normal still water had identical levels of cardiomyocyte proliferation when placed in the flume under static water conditions, with ∼2% of cardiomyocytes demonstrating IdU incorporation and CldU incorporation in response to this low level of stress (Fig. 6C). Although the 21-month-old control fish raised in normal still water aquarium conditions had exhibited a fall in proliferation after the high-intensity swimming protocol (Fig. 5C, Fig. 6D), their 21-month-old siblings raised in running water were able to maintain their levels of cardiomyocyte proliferation (∼2%) under this challenge. This was the same level of proliferation as seen in their siblings within the static arm of the experiment. Despite this improved response to an intense exercise challenge, all 21-month-old fish, regardless of rearing or experimental conditions, contained 25% dual-labelled cycling cardiomyocytes, suggesting this finding was dependent on age rather than environmental conditions (Fig. 6E,F). Similarly, apoptotic indices remained unchanged, with low levels of programmed cell death occurring in all groups at this age (Fig. 6G). Post-mortem measurements indicated there was no change in overall somatic length (Fig. 7A) or cardiac ventricle length (Fig. 7B). Finally, we examined the ventricular myocardial architecture using midline sections of these hearts stained with Masson's trichrome. This revealed increased levels of fibrosis throughout the myocardium in the zebrafish raised in running water compared with their sibling controls (Fig. 7C,D). Thus, although long-term exercise produced fitter fish, with better preserved cardiomyocyte replicative response under high-intensity exercise, this was at the cost of accelerated myocardial fibrosis.

## DISCUSSION

### Zebrafish as a model of human aging

In this study, we have shown that zebrafish can be used to study aspects of normal human cardiovascular aging, as our studies indicate that zebrafish follow the human Hamiltonian aging pattern, in which there is an exponential increase in the ageing phenotype after sexual maturity (Cohen, 2018), rather than indeterminate growth (Singleman and Holtzman, 2012; Gerhard et al., 2002). After reviewing papers that had previously suggested indeterminate growth, we note that the data are similar to ours, but conclusions had been drawn from regression lines. Both our data and that of Singleman and Holtzman (2012) also show a linear relationship with body length and ventricular length. However, although the Zhu group (Zhou et al., 2020) also demonstrated the same ratio as us, 0.4 mm cm^−1^ at 24 and 36 months, it was 0.3 mm cm^−1^ from 6 to 12 months. Thus, overall, the 6-month-old fish represent the growth phase of young sexually mature individuals, the equivalent of humans in their second decade, and the 21-month-old fish represent the post-reproductive midlife phase after ∼50 years (Cohen, 2018). Architectural changes within the heart that occur in human middle-old age, such as myocardial fibrosis (Lakatta, 2002), were only seen after 21 months in our studies and support this chronological equivalence. The extension of epicardial fat across the heart is also a sign of aging (Obas and Vasan, 2018) but may be exuberant because of nutritional excess in the laboratory environment (Faillaci et al., 2018).

Underlying this constrained growth pattern in the zebrafish heart is continuous low-level cardiomyocyte proliferation, which remains throughout adult life, as also observed in the human heart (Becker and Hesse, 2020). The cardiac metrics of ventricular length and compact myocardium thickness, measured in this study, support progressive growth of the heart under this constant proliferation, but it is the emergence of increased programmed cell death from 18 months that appears to halt overall organ growth. It seems likely that the escalating level of apoptosis at 24 months and beyond leads to an eventual net loss of cardiomyocytes and compensatory replacement fibrosis required to maintain myocardial integrity. Similar increases in programmed cell death occur in the aging rat heart (Ko et al., 2013), and have also been noted in the aging human heart (Keller and Howlett, 2016). The caspase 3 labelling we found indicates the occurrence of increased levels of cell death. However, as we cannot factor the temporal period for apoptosis to occur relative to the time of cell cycle, it is not possible to make exact mathematical calculations of the changes in myocardial number. Moreover, as cells are lost, there may not necessarily be an immediate reduction in myocardial volume, as the entirety of the ventricle provides a scaffold structure. Compensatory enlargement of remaining cardiomyocytes may be possible, but this would be only 1-2% by volume and not readily detected by these analyses.

In this study, we have tried to evaluate cardiac performance and reserve in fish of differing ages. As with human studies, it is difficult to directly assess cardiovascular function without also assessing the musculoskeletal system in some way. Voluntary swimming activity, which was slower in the 18-month-old fish than in the 6-month-old fish, is a similar test to the human 6-min walk test (Giannitsi et al., 2019). Although such voluntary activity testing can expose basal physiological limitations – cardiovascular or musculoskeletal – it can also reflect differences in behaviour. A fuller assessment of cardiac capacity requires engagement of all latent cardiac reserve. This normally requires physical exercise, such as a treadmill or exercise bicycle test, and presumes physical strength and muscle endurance are not limiting factors (Corra et al., 2018). We constructed the Ucrit test to assess this. Within the protocol, the zebrafish were exposed to initial speeds and time intervals that required sustained aerobic, rather than anaerobic, sprinting activity, and are therefore unlikely to limit the fish because of acute muscular fatigue. Hence, the reductions in spontaneous activity in older fish, supported by the analysis of critical swimming speed, is strong evidence of an inability to perform aerobic exercise. These results confirm and extend the findings of previous studies (Philpott et al., 2012). However, when taken together with the *nppa* expression data and the progression to adverse myocardial remodelling, our studies strongly implicate current myocardial stress and impending cardiac insufficiency. Both *nppa* and *nppb* have been previously reported to be elevated in very aged zebrafish hearts (Sun et al., 2015) but in our studies only *nppa* transcripts levels were raised, with *nppb* levels unaffected at 20 months, around the time when swimming speed is reduced but before there are marked increases in cardiomyocyte cell death or fibrosis. This differential activation is likely to be because the *nppa* gene is more sensitive to cardiac stress than the *nppb* gene (Man et al., 2018), and hence in our studies we consider this to indicate impending rather than fully established cardiac decompensation, as reported in other studies carried out in older fish (Sun et al., 2015). Importantly, further studies specifically to examine myocardial function, such as Langendorff chamber studies or echocardiography, might be required to examine cardiac function in more detail.

Having established this age-related deterioration in cardiac performance and corresponding changes in basal cardiomyocyte turnover, we wondered whether exercise would invoke a cardiomyocyte hyperplasia response. The 72-h intense exercise regimen involved a water speed of 3 BL sec^−1^, close to the critical swimming speed of the 21-month-old fish but low enough for them to complete the protocol without failing, and for younger fish it still proved a sufficient physiological stimulus for cardiomyocyte replication. This level of prolonged cardiovascular stress could be likened to the stress from a prolonged life-threatening human episode, such as critical sepsis, COVID-19 infection, major surgery, trauma or the loading of non-ischaemic cardiomyocytes during myocardial infarction. It was interesting to note that the flume environment itself, in the absence of water flow, initiated a robust proliferative response in the older fish, and the subsequent reduction in proliferation under the flowing water conditions therefore suggests exhaustion of available replicative capacity. It would be interesting to know whether there were long-term myocardial consequences of this brief intense exercise. However, owing to the experimental design with immediate culling of fish to analyse cell turnover, it was not possible to do this.

Cardiomyocyte loss underlies heart failure in aging humans, and an active lifestyle with regular exercise is generally considered to be of benefit. We were able to model the activity by housing zebrafish in tanks with running water. In contrast to other similar studies of long-term exercise (Jean et al., 2012), there was no change in the size of the hearts in our fish, probably because the exercise in our studies was less extreme. However, the increase in Ucrit indicated improved aerobic exercise capacity and was of a level similar to that when zebrafish were repeatedly made to swim to exhaustion (Gilbert et al., 2014). When these active fish were given the 72-h exercise challenge, they were protected from the fall in cardiomyocyte proliferation exhibited by their siblings raised in static water, although they still exhibited an increase in proliferation on entering the flume under non-running conditions. This improved response to a strong physiological challenge does appear to be at a cost to overall myocardial architecture as histological staining indicated accelerated fibrotic changes.

Zebrafish are notable for their ability to regenerate many of their organs after injury. Although most regeneration studies are generally carried out in zebrafish younger than 6 months of age (Itou et al., 2012), a period in which somatic growth is responsive to stocking and food conditions (Wills et al., 2008), there is no difference in the potential for fin or heart regeneration between young or aged fish (Itou et al., 2012). Hence, it seems unlikely that the effects of aging we have described are due to a late onset failure of cardiac regenerative response. However, in these experiments, we have identified two different patterns of proliferation. Some cardiomyocytes undergo a single round of replication, indicated by thymidine analogue incorporation either at the start or at the end of the exercise period, but there are other cardiomyocytes that incorporate thymidine analogues delivered 72 h apart. In younger fish, the single-cell cycle behaviour is predominant, with some increase in dual-labelling cells under proliferative stress. Interestingly, 25% of proliferating cardiomyocytes in older fish, irrespective of being raised in static or running water or the degree of proliferative stress, are dual labelling. It seems unlikely that vertebrate cells would remain in S phase for 72 h, and so this likely reflects progression within two replicative events. Further studies will be required to determine whether this is continuous cycling or whether this represents a population of cardiomyocytes with stem-like behaviour undergoing rounds of asymmetric cell division. In any event, the high percentage of cells with this behaviour in older fish compared to young fish suggests they are either a reserve or residual population forced into activity under minimal stress conditions in aged fish, or that the dual labelling represents a smaller population of cardiomyocytes capable of entering cell cycle that must continue to cycle to meet the imposed demand.

Taken together these studies indicate that occult changes in the pattern of cardiomyocyte turnover underly the overt myocardial aging phenotype. Most obviously, compensatory fibrosis occurs when elevated levels of programmed cell death outstrip baseline cardiomyocyte proliferation. However, more subtly, the proliferative profile of the cardiomyocytes also changes from a normal pattern of replicating cardiomyocytes entering and then leaving cell cycle, to an aged pattern in which 25% of replicating cardiomyocytes continuously cycle. Although the aged pattern of recycling cardiomyocytes does not change depending on the long-term exercise status of the fish, these cells may be more robust under severe stress. The price to be paid, however, is accelerated myocardial fibrosis. It is not clear whether the increased fibrosis eventually leads to greater impairment of diastolic heart function or a greater risk of arrythmia. Human studies that imposed exercise in aging have yielded results similar to our own zebrafish findings, showing that the exercise makes the subjects fitter but does not change the trajectory of their cardiac aging (Jakovljevic et al., 2015). Recent studies have indicated a close association between fibrosis and early onset atrial fibrillation (Elliott et al., 2018). It will be important to ensure that the general reduction in morbidity and mortality that exercise brings outweighs any increased morbidity and mortality from fibrosis when advocating different forms of exercise in later adult life. More pertinently, this study has relevance to systemic illnesses that, although not directly affecting the heart, places the cardiovascular system under increased stress. These impaired cardiomyocyte proliferative responses in older adults that have not remained active in later life may underlie the increased mortality and morbidity during and after illnesses, such as COVID-19, and is an area potentially amenable to pharmacological intervention.

## MATERIALS AND METHODS

### Animals

Wild-type zebrafish (AB strain) were maintained under standard conditions (Westerfield, 1993) in a 14/10 h light/dark cycle and fed with commercial flake food supplemented with brine shrimp. They were housed in groups of 20 fish in 12-litre holding tanks within a circulating aquarium system (Aqua Schwartz, Germany). The animals were held under the Animals (Scientific Procedures) Act 1986, United Kingdom (project licence PPL6004548), and conformed to Directive 2010/63/EU of the European Parliament. All experiments were approved by the Newcastle University Animal Welfare and Ethical Review Board.

### Histological staining

Hearts were dissected, following terminal anaesthesia with ethyl 3-aminobenzoate methanesulfonate and euthanasia by destruction of the brain, and briefly incubated in Ringer's solution containing 10 mM calcium chloride to effect ejection of blood. Hearts were then fixed in 4% paraformaldehyde for 24 h at 4°C, embedded in paraffin wax, and 8 µm sagittal sections were prepared. De-waxed sections were incubated overnight in Bouin's fixative (VWR, UK) and then stained using a Masson's trichrome reagent kit (Sigma-Aldrich, UK) according to manufacturer's instructions. Fat and fibrosis scoring was independently carried out by L.B.M. and D.J.H., who during the analysis were blinded with regard to the experimental groups and each other, yet produced identical scores.

### Immunohistochemistry and image analysis

Individual zebrafish were exposed to either IdU (250 mg l^−1^) or CldU (250 mg l^−1^) in 200-ml vessels containing normal aquarium water for 24 h. Hearts were collected as above and 8-µm sections obtained. After antigen retrieval with heated 10 mM citrate buffer, nuclear DNA was partially digested with 2N HCl for 30 min. Incorporation of each thymidine analogue was identified using antibodies specific to IdU (1:200, mouse anti-BrdU, BD Biosciences, 347580) and CldU (1:250, rat anti-BrdU, Abcam UK, ab6326). Cardiomyocytes were identified using an antibody against myocyte-specific enhancer factor 2 (1:50, rabbit anti-Mef2, Santa Cruz Biotechnology, USA, sc-313). To identify cells undergoing programmed cell death, sections were prepared as before with antigen retrieval in heated 10 mM citrate buffer. Sections were probed with rabbit anti-cleaved Cas3 (1:100, Cell Signaling Technology, USA, 9661), and cardiomyocytes were identified with mouse anti-MF20 antibody (1:400, Developmental Studies Hybridoma Bank, USA). All primary antibodies were reported using Alexa Fluor-tagged secondary antibodies (Life Technologies, UK) and mounted using medium containing DAPI. Images were captured on a Zeiss Axioplan II epifluorescence microscope (Zeiss, Germany) and a minimum of four sections at least 60 µm apart were analysed using Fiji/ImageJ (Schindelin et al., 2012). Counting was performed manually, ensuring that only cardiomyocyte nuclei were examined for IdU^+^, CldU^+^ or Cas3^+^ labelling. Image analysis and counting was initially performed by D.B. and then repeated independently by L.B.M. Experiments in which the immunolabelling had failed and there was no specific labelling, were excluded.

### Quantitative RT-PCR

Total RNA was extracted from four single whole hearts from each experimental group using TRIzol reagent (Life Technologies, UK) and cDNA was synthesised using a high-capacity cDNA reverse transcription kit (Life Technologies, UK). Real time quantitative PCR was performed in triplicate, and a mean value of the technical replicates was obtained using SYBR green (Life Technologies, UK) with a 7500 Real Time PCR system (Life Technologies, UK) and SDS software (Life Technologies, UK). Primers used were as follows: nppa fwd, 5′-GCAACATGGCCAAGCTCAAG-3′ and rev, 5′-CTGTCCCAGGATGTGGAAGG-3′; nppb fwd, 5′-CATTCCCGCTTCAAAGCACA-3′ and rev, 5′-CTTCTCTTTCCGCCGGTGTT-3′; and gapdh fwd, 5′-TCACACCAAGTGTCAGGACG-3′ and rev, 5′-TCAAGAAAGCAGCACGGGTC-3′. Relative expression levels were then compared using the 2−ΔΔCt method (Livak and Schmittgen, 2001).

### Spontaneous swimming

Single zebrafish were placed in a 3-litre transparent tank (surface area 200 mm×150 mm) filled with aquarium water to 50 mm depth over a piece of white paper. Zebrafish were allowed to acclimatise to the environment for 5 min and then three 1-min videos were captured at a rate of 25 frames per second (ImperX VGA 210 CCD camera with 12.5 mm lens, Lynx GigE software; Multipix Ltd, UK). The path of the fish was analysed in Fiji/ImageJ using the MTrack2 plug-in (Schindelin et al., 2012). Average speed was calculated by measuring total distance travelled/total time and, after measuring the length of each zebrafish, converted to body lengths per second (BL sec^−1^).

### Critical swimming speed

The flume for the assessment of Ucrit and high-intensity swimming was fabricated from a 6-mm transparent acrylic sheet to provide a main tank with the following internal dimensions: length 950 mm×width 50 mm×height 80 mm. Within this, an insert was introduced with a central channel with a cross-sectional area width of 18 mm×length 495 mm and a height of 78 mm, which allowed a higher water speed to be achieved. Mesh barriers with 4 mm×4 mm holes were placed at the ends of the tanks, and an acrylic lid to prevent escape of fish. Flow was provided by a Fluval 406 canister filter containing an impeller pump (Hagen, Germany). The speed of water within the chamber was monitored using a flow meter (Digimeter) within the inlet pipe, calibrated using polystyrene balls to measure water speed within the insert itself. Analysis of Ucrit was performed according to the schedule shown in Fig. 5A.

### High-intensity swimming

After removal of the Ucrit insert, the speed of water within the flume was adjusted to 3 BL sec^−1^, and groups of no more than 12 zebrafish of the same age and length were placed in the swim tunnel for 72 h. The water flows were reduced to allow feeding, and filters within the pump maintained water quality. An identical flume without flow was used to provide static water conditions for siblings. Experiments were performed as indicated in Fig. 5.

### Running water

When breeding zebrafish for experiments at 21 months of age, 40 zebrafish siblings were divided between two identical holding tanks. From 10 months of age, one of the tanks was provided with flowing water via a small impeller-driven aquarium filter (AP-650L, Hidom) that ejected a jet of water across the central portion of the holding tank. The filter pump was checked daily to ensure optimal function and the water stream developed by the pump was shown to be maximally 2 BL sec^−1^ in the centre of the tank by measuring the movement of polystyrene balls introduced into the water flow. Zebrafish raised in the normal aquarium water conditions were used for the experiments indicated in Fig. 5, and siblings raised in running water were used in the experiments shown in Fig. 6. This design ensured that a minimum number of animals were used.

### Statistics

Statistical analysis was carried out using GraphPad Prism 9 (GraphPad Software, USA). For all experiments, normality of data distribution was assessed and non-parametric testing carried out as appropriate. One- or two-way ANOVA was carried out for normally distributed data and non-parametric ANOVA was used for data that was not normally distributed (Kruskal–Wallis test), as indicated in the figure legends. Where there were only two groups, Student's *t*-test was employed after examining for normally distributed data (D'Agostino-Pearson normality test) and visual assessment of variance. Equal variance was confirmed by *F*-test. Statistical biological replicates (*n*) are indicated in figures.
